# Rapid and sustainable self-questionnaire for large-scale psychological screening in pandemic conditions for healthcare workers

**DOI:** 10.3389/fmed.2022.969734

**Published:** 2023-01-11

**Authors:** Carolina Soledad Romero, Maria Otero, Manuel Lozano, Carlos Delgado, Ana Benito, Juan Catala, Adina Iftimi, Jose De Andres, Markus M. Luedi

**Affiliations:** ^1^Anesthesia, Critical Care and Pain Department, Hospital General Universitario de Valencia, Valencia, Spain; ^2^Research Methodology Department, Universidad Europea de Valencia, Valencia, Spain; ^3^Preventive Medicine and Public Health, Food Sciences, Toxicology and Forensic Medicine Department, Universitat de Valencia, Valencia, Spain; ^4^Epidemiology and Environmental Health Joint Research Unit, Foundation for the Promotion of Health and Biomedical Research of Valencia Region, Universitat Jaume I-Universitat de València, Valencia, Spain; ^5^Department of Psychiatry, Valencia University General Hospital, Valencia, Spain; ^6^Department of Statistics and Operations Research, University of Valencia, Valencia, Spain; ^7^Department of Anaesthesiology and Pain Medicine, Inselspital, Bern University Hospital, University of Bern, Bern, Switzerland

**Keywords:** COVID-19, SARS-CoV-2, models, psychological, healthcare workers, stress, structural equation model, sustainability

## Abstract

**Background:**

The pandemic caused by a coronavirus (COVID-19) has shocked healthcare systems worldwide. However, the psychological stressors remain unclear. The objective of this study was to assess the impact of a major pandemic on healthcare workers. We hypothesized that exposure to the virus would be the primary cause of psychological stress perceived by healthcare workers.

**Methods:**

A national cross-sectional study conducted *via* an online questionnaire was distributed between April 9 and April 19, 2020 with a non-probabilistic sample technique. A structural equation model (SEM) was built with the variable “exposure to the virus” and the Psychological Stress and Adaptation at work Score (PSAS). “Exposure to the virus” was defined as the combined factors of ‘personal-sphere’, “work-related stress” and “hospital characteristics.” A generalized linear model (GLM) was also tested.

**Results:**

A total of 2,197 participants filled in the questionnaire and were analyzed. The exploratory factor analysis showed statistically significant variables related to the personal-sphere, work-related stress and the hospital’s characteristics, although the confirmatory factor analysis showed only the work-related stress factors to be significant. The GLM showed that personal-sphere-related variables (*P* < .001), stress at work (*P* < 0.001) and age (*P* < 0.001) were statistically significant.

**Conclusion:**

Physical exposure to the virus is an essential factor that contributes to the psychological impact perceived during the pandemic by healthcare professionals. A combination of personal-sphere variables, work-related stress and hospital characteristics is a significant factor correlating with the degree of stress measured by PSAS, a new and fast instrument to assess stress in healthcare workers.

## Introduction

Severe acute respiratory syndrome coronavirus 2 (SARS-CoV-2) has had a huge impact on society. Economic and political challenges have arisen, and individual and collective consequences are severe worldwide. Besides this unprecedented circumstance, many countries are facing an exigent healthcare system transformation (1).

Changing working conditions and unfamiliar roles were implemented so that extended care plans could cover pandemic novel needs. Fear of unavoidable exposure also aggravated the situation impairing mental health conditions, including increasing the risk of self-harm and suicide among healthcare workers (2). Many efforts in the clinical field of COVID-19 have been reported, but mental health has also been at stake during the outbreak. The stress experienced affected both the general population (3) and healthcare workers (4), who were among the most vulnerable groups. Information on the mental health impact of healthcare workers is still limited in European countries.

We aim to describe the psychological stress experienced by healthcare workers secondary to exposure to the SARS-CoV-2 pandemic in Spain. For this, the main objective of this study is to validate an instrument designed to assess the Psychological Stress and Adaptation at work Score (PSAS*).* This fast and self-reported questionnaire could potentially fill a gap in psychological evaluation in the era of new technologies and extraordinary situations like the coronavirus crisis, as well as play a major role when time for assessment is limited. Timely delivery of knowledge of the mental health impact in pandemics is crucial for healthcare leaders and policymakers to establish a Mental Health Crisis Response.

## Materials and methods

### Design and participants

This national, cross-sectional survey was performed by the Research Institute of the University General Hospital of Valencia, which was the coordinating center for the Psychological Impact of Coronavirus (PSIMCOV) network in Spain. The institutional ethics board of the University General Hospital of Valencia approved this study. A digitally signed informed consent form was embedded at the beginning of the survey and was a prerequisite for participating.

### Instrument

For the stress and psychosocial impact evaluation, PSAS, a 23-item questionnaire was used (Supplementary Data Sheet 1: questionnaires A, B, C, and D). The survey was preceded by demographics and general information questions (5, 6). Data on age, area, working environment, medical specialty, previous experience, and exposure to the virus were registered (Supplementary Data Sheet 1: items 2–23). *PSAS* is a combined measure of the scores obtained in four modified versions of validated psychological assessment tests : *(A) Healthcare Stressful Test* for identifying stressing factors at work (7, 8), *(B) Coping Strategies Inventory* for assessing problem-solving, self-criticism, emotional expression, willing thoughts, problem avoidance and social support spheres (9, 10), *(C) Font-Roja Questionnaire* for assessing satisfaction, pressure, relationships, relaxation, adequacy, control and task variety at work (11, 12), and *(D) Trait Meta-Mood Scale* for assessing interpersonal aspects of emotional intelligence (13, 14). Condensed versions of these tests had to be created to match a context in which participants had little time to complete them. At least one question of the validated translation was included for every dimension.

### Data collection

A non-probabilistic sampling technique was used and the questionnaire was distributed *via* email to national healthcare societies, professional organizations and the main social network platforms amongst healthcare professionals. Results were retrieved anonymously in an online database. The study was conducted during the most epidemiologically stressful stage so far of the emergency in Spain (between 9 and 19 April, 2020).

### Statistical analysis

**FIGURE 1:**
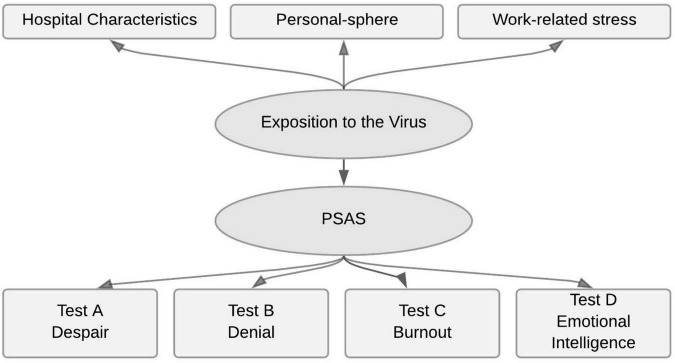
Structural equation model constructions. Psychological stress and adaptation at work score (PSAS).

Data were analyzed using the statistical software R (15) and the lavaan package (16). Only complete cases (*n* = 2,197) were included in the analyses. An exploratory structural equation model (SEM) was developed to investigate the associations between the stress levels measured by using PSAS and a theoretical exposure to the virus latent variable. The hypothesis was that exposure to the virus can predict the degree of psychological stress experienced at work (evaluated with the items of modified questionnaires A, B, C, and D in Supplementary Data Sheet 1). Regarding exposure to the virus (evaluated with items 5–22 of Supplementary Data Sheet 1), three latent variables were considered: “personal-sphere” (perceived consequences of the pandemic at the personal level, included personal exposure to the virus), “work-related stress evaluation” (perceived consequences of the pandemic at the workplace, including work exposure to the virus) and “hospital characteristics” (workplace characteristics). This construct was inferred according to the hypothetical model shown in Figure 1. The “personal-sphere latent variable” was built by factor analysis from five measured indicators with higher scores corresponding to situations associated with more stress: living with dependents (item 9); living with your partner (item 10); poor balance between family and work (item 21); need for psychotherapy (item 22); and fear of the coming economic crisis (item 20). Using the same criteria, the latent variable “work-related stress evaluation” was built by grouping seven observed variables: Personal exposure to the virus (item 12), physical work-related overload (item 13), psychological work-related overload (item 14), perception of risk exposure at work (item 15), psychosocial work environment distress (item 16), strict hierarchy at work (item 17), and distress due to new circumstances (item 18). The “hospital characteristics” latent variable was measured as: Primary Hospital, Secondary Hospital, Tertiary Hospital, General Practitioners in Medical Centres and Ambulance Services (items 7 and 11). Hospital, General Practitioners in Medical Centres and Ambulance Services. Exposure to the virus was then weighed with the PSAS instrument, which was created by combining the four condensed versions of the validated tests described above; Test A = “Despair”; Test B = “Denial”; Test C = “Burnout”; and Test D = “Emotional intelligence.”

Several authors extended a recommendation in the evaluation of goodness of fit with several indexes (17, 18), such as that the Chi-Square values associated with a non-significant *p*-value indicate good model fit (19). Comparative fit index (CFI) values higher than 0.90 indicate acceptable model fit. The residuals for the standardized root mean square (SRMR) ≤0.08 indicate a good fit for the model. Root mean square error of approximation (RMSEA) values below 0.1 indicate good model fit and values ≤0.05 indicate a very good fit (17–20). To mitigate overfitting of the model, a cross-validation technique was carried out with a 70% sample from the study for the exploratory factor analysis (EFA) with a total of 1,538 respondents, and the remaining 30% (659 respondents) for the confirmatory factor analysis (CFA). Afterward, the proposed model was tested with the total sample for exploratory and confirmatory analysis. A generalized linear model (GLM) was also built-in to assess the direct effect of the “hospital characteristics” factors and personal-sphere and work-related stress evaluation with PSAS scores, adjusting for age and societal impact on the staff.

## Results

In total, 2,253 surveys were completed and referred to the coordinating center from April 9 to April 19, 2020. A total of 45 respondents had incomplete answers and 11 surveys did not comply with the quality definition. Thus, the final analysis collected data from 2,197 healthcare workers.

**FIGURE 2:**
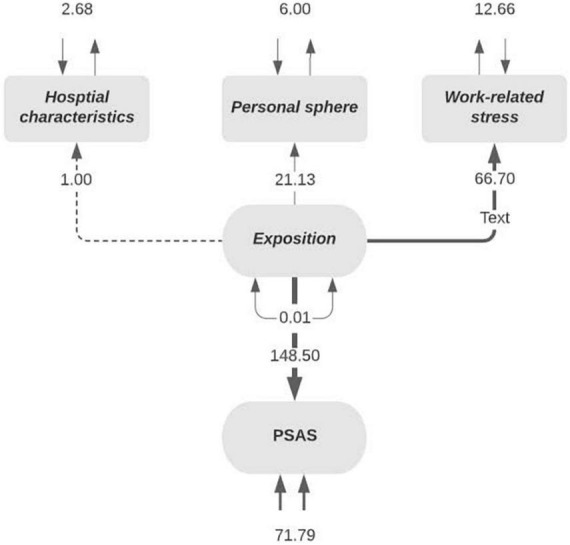
Exposition to the virus in healthcare workers during coronavirus (COVID-19) pandemic.

Demographics and the main characteristics of the participants can be seen in Table 1. A structural model was designed to estimate the relationship between the measured constructs and it is represented in Figure 2. Model fit was evaluated to ascertain compatibility between the model proposed and the empirical evidence obtained as described in the following equation:

**TABLE 1:** Basal characteristics of the subset sample for analysis.

Variable	Sample 70% (*n* = 1,549)		Sample 30% (*n* = 648)		*P*-value
	*n*	%	*n*	%	
**Hospital characteristics**	–	–	–	–	–
Primary hospital	817	52.7	346	53.4	n.s
Secondary hospital	125	8.1	63	9.7	–
Tertiary hospital	106	6.8	45	6.9	–
General practitioners in medical centers	190	12.3	85	13.1	–
Others	311	20.1	109	16.8	–
**Dependents**	–	–	–	–	–
No	845	54.6	342	52.8	n.s
Yes	704	45.4	306	47.2	–
**Partner**	–	–	–	–	–
No	375	24.2	154	23.8	n.s
Yes, but non-health worker	784	50.6	323	49.8	–
Yes, health worker	390	25.2	171	26.4	–
**Psycotherapy**	–	–	–	–	–
No	1210	78.2	515	79.6	n.s
No, but I would like to start	245	15.8	85	13.1	–
Yes, before_the_crisis	57	3.7	34	5.3	–
Others	36	2.3	13	2	–
**Relatives**	–	–	–	–	–
No	1199	77.4	499	77	n.s
Yes	350	22.6	149	23	–
Age	44.453	11.39	44.767	11.489	n.s
Hierarchy at work	2.335	2.621	2.238	2.662	n.s
Physical overload	2.668	1.622	2.753	1.665	n.s
Psychological overload	3.192	1.503	3.241	1.513	n.s
Risk perception exposure	3.633	1.491	3.676	1.447	n.s
Dynamics distres	2.52	1.599	2.52	1.56	n.s
Hierarchy distress	2.362	1.746	2.35	1.767	n.s
New circumstances distress	2.33	1.676	2.29	1.669	n.s
Personal society impact	1.179	1.499	1.227	1.5	n.s
Fear post crisis	3.399	1.615	3.36	1.654	n.s
Balance family work	3.046	1.718	3.048	1.76	n.s
Personal sphere	8.236	3.063	8.227	3.043	n.s
Work sphere	17.276	6.792	17.39	6.745	n.s
**COVID exposition**	**0.571**	**0.789**	**0.56**	**0.774**	**n.s**
Test A despair	5.364	2.473	5.452	2.496	n.s
Test B denial	15.966	6.737	16.082	6.529	n.s
Test C burnout	14.411	6.573	14.384	6.466	n.s
Test D emotional intelligence	6.156	3.723	6.144	3.73	n.s
**PSAS**	**41.896**	**15.535**	**42.062**	**15.026**	**n.s**

*P*-values are calculated with the Chi square test for proportions and the *t*-test for continuous variables.

Hospital characteristics: work setting of the healthcare worker undergoing the questionnaire; Dependents: a person in charge of children or dependents relatives; Partner: type of work of your partner; Psychotherapy: having received or receiving psychotherapy at the time of the assessment; Relatives: relatives with COVID-19 diagnosis while completing the questionnaire; COVID exposition: assessment of the degree of perceived exposition to the disease.

Exposition = ∼ 1 * hospital characteristics + personal sphere

*+ work related stress*

*PSAS ∼ Exposition*

*hospital characteristics ∼∼ hospital characteristics*

*personal sphere ∼∼ personal sphere*

*work related stress ∼∼ work related stress*

The exploratory analysis for the subset model with the 70% sample showed a very good fit for the data with a Chi-square value obtained for the SEM analysis of *P* = 0.141. Other indexes obtained were the goodness of fit index (GFI = 0.999), the comparative fit index (CFI = 0.95), the standardized difference between the observed correlation and the predicted correlation (SRMR = 0.009), the expected cross-validation index (ECVI = 0.013) and the RMSEA, with a value of 0.025 and a confidence interval (CI, 0.001–0.061) of *P* = 0.849, which does not reject the null hypothesis; i.e., the model is the right fit for the data. The latent variables which were statistically significant were “hospital characteristics” (*P* = 0.001), “personal-sphere” (*P* = 0.005), and the variance (*P* = 0.001), “work-related stress” (*P* = 0.004), and the variance (*P* = 0.001) and PSAS (*P* = 0.001). The regression analysis amongst PSAS and “exposure to the virus” was statistically significant too (*P* = 0.004). The confirmatory analysis with the remaining 30% of the sample showed that only the variable “hospital characteristics” was statistically significant (*P* = 0.001). The results of the exploratory and confirmatory analysis of the subset are shown in Table 2. Later, the construct model was tested in the whole sample with a Chi-square value obtained for the SEM analysis of *P* = 0.837. Other indexes obtained were the goodness of fit index (GFI = 1), the comparative fit index (CFI = 0.0.998), the standardized difference between the observed correlation and the predicted correlation (SRMR = 0.004), the expected cross-validation index (ECVI = 0.011) and the RMSEA, with a value of.031 CI (0.005–0.060) *P* = 0.0836. The variables statistically significant for the exploratory analysis were the variance for “hospital characteristics” (*P* = 0.0001), the latent variable “personal-sphere” (*P* = 0.023), and the variance (*P* = 0.001), “work-related stress” (*P* = 0.023), and the variance (*P* = 0.001) and PSAS (*P* = 0.001). The regression analysis among PSAS and “exposure to the virus” was statistically significant as well (*P* = 0.002). In the confirmatory analysis, the statistically significant variable was the “work-related” stress factors (*P* = 0.001).

**TABLE 2:** Results of the exploratory factor analysis (EFA) and confirmatory factor analysis (CFA).

	Exploratory factor analysis (*n* = 2,197)			
	Estimate	Standard error	*Z*-value	*P*-value
**Latent variables**	–	–	–	–
Exposition = ∼	–	–	–	–
Hospital characteristics	1.000	–	–	–
Personal-sphere	21.133	9.319	2.270	0.023
Work-related stress	66.698	29.339	2.273	0.023
Regressions	–	–	–	–
PSAS∼	–	–	–	–
Exposition	148.495	65.318	2.273	0.023
**Variances**	–	–	–	–
Hospital characteristics	2.680	0.081	33.127	0.001
Personal-sphere	6.002	0.203	29.615	0.002
Work-related stress	12.663	0.982	12.895	0.001
PSAS	71.785	4.980	14.415	0.001
Exposition	0.007	0.007	1137	0.255
	**Confirmatory factor analysis (*n* = 2197)**			
	**Estimate**	**Standard error**	***Z*-value**	***P*-value**
**Latent variables**	–	–	–	–
Exposition = ∼	–	–	–	–
Hospital characteristics	1.000	–	–	–
Personal-sphere	34.889	27.331	1.277	0.202
Work-related stress	87.616	77.720	1.127	0.260
**Variances**	–	–	–	–
Hospital characteristics	2.684	0.081	33.123	0.000
Personal-sphere	5.136	3.499	1.468	0.142
Work-related stress	19.396	22.051	0.880	0.379
Exposition	0.003	0.005	0.689	0.491

PSAS: Psychological stress and adaptation at work score.

The second tested model was the GLM model to explore the variable “hospital characteristics.” The model fitted was:

*PSAS ∼ hospital characteristics + personal sphere + work*

*related stress + social impact + age*

Working in the ambulance services was statistically significant (*P* = 0.003), while working in a secondary hospital (*P* = 0.068), working in a tertiary hospital (*P* = 0.052) and working as a general practitioner (*P* = 0.062) were not significant. In this model, the other variables, such as the personal-sphere (*P* < 0.001) and work-related stress (*P* < 0.001), were also statistically significant. The r^2^ for the GLM obtained was 0.531 but when “age” and the evaluation of the social situation to which the healthcare workers are exposed were taken into consideration with an increase of the r^2^ up to 0.572. Both variables of age (*P* < 0.001) and the social situation experienced by the healthcare workers due to COVID-19 (*P* < 0.001) were statistically significant. The results of the GLM analysis are described in Table 3.

**TABLE 3:** Results of generalized linear model (GLM) for the sample.

	GLM analysis (*n* = 2,197)			
	Estimate	Standard error	*t*-value	*P*-value
(Intercept)	16.711	1.154	14.482	<0.001
Hospital characteristics	–	–	–	–
Secondary hospital	0.848	0.795	1.067	0.286
Tertiary hospital	0.065	0.875	0.074	0.941
General practitioners in medical centers	1.956	0.683	2.865	0.004
Ambulance services	1.89441	0.578	3.280	0.001
Personal sphere	0.82466	0.082	10.024	<0.001
Work-related stress	1.35866	0.037	36.582	<0.001
Societal impact	1.54405	0.146	10.601	<0.001
Age	-0.171	0.019	-8.857	<0.001

## Discussion

We have constructed an equation model to validate a new instrument to measure psychological impact in times of crisis among healthcare workers in Spain. Questionnaires for psychological evaluation in healthcare workers during emergencies are often used but hardly validated in the clinical setting. To the best of our knowledge, this is the largest study on healthcare workers to use a structural equation model to assess the psychological stress perceived during the COVID-19 pandemic.

The dynamics of stress generation during a healthcare crisis are not well-described. A Cochrane systematic review (21) studying the effects of burnout and occupational stress in healthcare workers concluded that the level of evidence from the random clinical trials was low regarding the effect of selected interventions in reducing stress, due to the small sample size of the studies available. Although burnout and occupational stress are highly prevalent among medical doctors, the quality of research examining the benefits of psychosocial/behavioral interventions remains low (22). Despite increased scientific attention, no structured instrument has been proven to be reliable in assessing stress in healthcare workers and during healthcare crises.

The results reveal a relationship between the perception of exposure to the virus and the “‘personal-sphere,” “work-related stress factors,” and “hospital characteristics.” Several previous studies have examined aspects of psychological stress from various perspectives (23, 24). Consistent efforts have been made to determine stress experienced by healthcare workers and to establish how to deal with it effectively (25). The present study has identified the potential importance of exposure to the virus as a construct of several external factors, including personal factors, work-related stressors, and characteristics of the workplace, as an important element within the context of psychological stress in healthcare workers.

We have introduced two models in relation to psychological impact: a SEM model and a GLM model. The perceived stress level is present and predominant in workers who come in direct contact with COVID-19 patients. The major external risk factors involved in the psychological impact are personal exposure to SARS-CoV-2. This fact has been confirmed previously in other studies that evaluated healthcare workers dealing with critical situations (26, 27). The personal-sphere included variables such as the employee’s work-family conflict between the family and work domains. This has previously been reported to influence individuals’ work performance (28) and work engagement. The work-related stress factors studied included physical and psychological overload and the system dynamics that arose from a novel situation of uncertainty and ever-changing protocols.

Validation of the PSAS questionnaire with a SEM model has been carried out. The selection and adaptation of validated questionnaire items has allowed the creation of this self-conducted questionnaire, which has shown to be valid to be implemented in special situations when time is scarce and resource allocation is limited. The questionnaire is completed in less than 10 min in every case, favoring a high response rate Several interventions have been described as providing mild to moderate relief of psychological symptoms, like meditation (29) and mindfulness in healthcare workers (30), particularly in nurses (31). Meditative interventions could help produce stress reduction daily and can be easily continued during crisis situations like the COVID-19 pandemic. West et al. (32) previously stated that “Physicians burnout has reached epidemic levels”; thus, it is paramount to promote the study of these external factors that might help in planning future organizational-based interventions and minimizing the stress experienced during pandemics by one of the most vulnerable groups in a major healthcare crisis. Further research is needed to establish which interventions are most effective, as well as which combination of organizational and individual solutions can deliver improvements in wellbeing and stress reduction interventions. Promoting long-term follow-up studies will help to define the theoretical framework and potential effects of these interventions.

### Implications to education

Mental health assessment is a very complex area that involves the comprehensive examination of many aspects of human life. In addition, the factors that affect mental health are many and varied. Better mental health has been related to people with more years of education (33), however, the healthcare population is a group that has a significant resistance to assessing and talking about mental health (34). Therefore, this quick questionnaire, which assesses many aspects of mental health in a compressed form, may be the first step in prompting a health care worker to seek psychological help. It can be filled out anywhere and takes no more than a few minutes, so each person can fill it out at a time that suits him or her best.

Finally, the major educational implications of this work are: (1) Providing specific self-assessment learning about the psychological effects during crisis, (2) Emphasis on the mastery of self-assessment even in adverse conditions, (3) Emphasis on thinking and reasoning about the environment affect us, and (4) Development of strategies to seek for help quickly.

### Limitations

This study has some limitations. First, this is an internet-based survey, and data were acquired *via* a self-conducted questionnaire. Yet, this is the largest study on healthcare workers assessing psychological stress perceived during the COVID-19 pandemic. Second, the critical nature of the Spanish situation did not allow for a previous assessment of stress levels in the population. The structural equation model provides some confidence. Third, reduced versions of the psychological tests were used to guarantee completion of an option successfully described before. Having four scales to assess PSAS with 45 questions, some of them could overlap. Fourth, the transversal nature of the study permits the existence of confounding factors that could not be identified. Fifth, more than 66% of the respondents were working in the second-least affected area in Spain, so the reported stress impact could be underestimated. Yet, this burden might be a global average.

## Conclusion

Exposure to the virus is one of the major factors influencing the stress perceived by healthcare workers. Other factors include personal-sphere variables, work-related stress factors and hospital characteristics. The PSAS questionnaire is a new and fast instrument to assess psychological stress in healthcare workers during a major crisis.

## Data availability statement

The raw data supporting the conclusions of this article will be made available by the authors, without undue reservation.

## Ethics statement

The studies involving human participants were reviewed and approved by Consorcio Hospital General Universitario de Valencia. Written informed consent for participation was not required for this study in accordance with the national legislation and the institutional requirements.

## Author contributions

CR participated in conception, design, analysis, and interpretation of the data, drafted and revised the manuscript critically for important intellectual content, and gave the final approval for the version to be published. MO, CD, JC, and JD participated in conception, design, and interpretation of the data, revised the manuscript critically for important intellectual content, and gave the final approval for the version to be published. AI and ML participated in design, analysis, and interpretation of the data, revised the manuscript critically for important intellectual content, and gave the final approval for the version to be published. ML and CR took responsibility for data accuracy and analytic and reporting integrity of the study. MML participated in the conception, design, and drafted and revised the manuscript. All authors contributed to the article and approved the submitted version.
