# Supplementary material for: Rapid and sustainable self-questionnaire for large-scale psychological screening in pandemic conditions for healthcare workers
Source: Front Med (Lausanne). 2023 Jan 11;9:969734. doi: 10.3389/fmed.2022.969734 (PMC9874091; doi:10.3389/fmed.2022.969734)
Supplement: Supplementary file 1 [file Data_Sheet_1.PDF]

PSAS

Psychological Stress and Adaptation at work Score

Welcome to this very important questionnaire with which we researchers want to asses the psychological stress in healthcare workers, and particularly in nurses.
Please note that the questionnaire might be anonymous or if you prefer, you might leave your name and the researchers will contact you for a second survey to evaluate the basal stress level.

1. **Your name:** ____________________

Questionnaire A: Modified Healthcare Stressful Factors Test

State how frequently the following situations have been stressful to you since the present emergency started.

1.1. I felt hopeless regarding to a patient not responding to treatment or suffering.
☐ Never. ☐ Sometimes. ☐ Often. ☐ Always.

1.2. I felt alone when a patient was in an emergency situation or dying.
☐ Never. ☐ Sometimes. ☐ Often. ☐ Always.

1.3. I did not know how to manage new or specialized equipment.
☐ Never. ☐ Sometimes. ☐ Often. ☐ Always.

1.4. I received negative comments from my colleagues.
☐ Never. ☐ Sometimes. ☐ Often. ☐ Always.

1.5. I have been in disagreement with task or resource management at work.
☐ Never. ☐ Sometimes. ☐ Often. ☐ Always.

Questionnaire B: Modified Font-Roja Satisfaction at Work Test

State how frequently the following situations have been stressful to you since the present emergency started.

1.1. I feel unsatisfied at work and poorly recognized.
☐ Never. ☐ Rarely. ☐ Sometimes. ☐ Often. ☐ Very often. ☐ Always.

1.2. I feel my workload is overwhelming and I do not have time to complete my tasks.
☐ Never. ☐ Rarely. ☐ Sometimes. ☐ Often. ☐ Very often. ☐ Always.

1.3. Social relationships feel unfulfilling to me.
☐ Never. ☐ Rarely. ☐ Sometimes. ☐ Often. ☐ Very often. ☐ Always.

1.4. I feel poorly remunerated for my job.
☐ Never. ☐ Rarely. ☐ Sometimes. ☐ Often. ☐ Very often. ☐ Always.

1.5. I do not feel properly trained for my job.
☐ Never. ☐ Rarely. ☐ Sometimes. ☐ Often. ☐ Very often. ☐ Always.

1.6. I feel unable to organize my time or to learn new things at work.
☐ Never. ☐ Rarely. ☐ Sometimes. ☐ Often. ☐ Very often. ☐ Always.

1.7. I feel my job is monotonous and repetitious.
☐ Never. ☐ Rarely. ☐ Sometimes. ☐ Often. ☐ Very often. ☐ Always.

Questionnaire C: Modified Coping Strategies Inventory

State how frequently the following situations have been stressful to you since the present emergency started.

1.1. I tried to avoid facing a problem.
☐ Never. ☐ Rarely. ☐ Sometimes. ☐ Often. ☐ Very often. ☐ Always.

1.2. I blamed myself for something that happened.
☐ Never. ☐ Rarely. ☐ Sometimes. ☐ Often. ☐ Very often. ☐ Always.

1.3. I avoided to express my feelings with my peers during or after the emergency.
☐ Never. ☐ Rarely. ☐ Sometimes. ☐ Often. ☐ Very often. ☐ Always.

1.4. I wished a situation not to be happening or not to have happened at all.
☐ Never. ☐ Rarely. ☐ Sometimes. ☐ Often. ☐ Very often. ☐ Always.

1.5. I had a bad reaction in the presence of other people to express contained emotions.
☐ Never. ☐ Rarely. ☐ Sometimes. ☐ Often. ☐ Very often. ☐ Always.

1.6. I chose not to analyze the causes of something that happened and avoided introspection so that I could keep doing my tasks.
☐ Never. ☐ Rarely. ☐ Sometimes. ☐ Often. ☐ Very often. ☐ Always.

1.7. I took action before thinking because I was feeling overwhelmed.
☐ Never. ☐ Rarely. ☐ Sometimes. ☐ Often. ☐ Very often. ☐ Always.

1.8. I avoided spending time with people from my environment and I isolated myself because of my personal distress.
☐ Never. ☐ Rarely. ☐ Sometimes. ☐ Often. ☐ Very often. ☐ Always.

Questionnaire D: Modified Trait Meta-Mood Scale Test

State how frequently the following situations have been stressful to you since the present emergency started.

1.1. I believe I do not think about my feelings very much.
☐ Never. ☐ Rarely. ☐ Sometimes. ☐ Often. ☐ Very often. ☐ Always.

1.2. I usually do not fully understand why I feel sad or why I think too much about things.
☐ Never. ☐ Rarely. ☐ Sometimes. ☐ Often. ☐ Very often. ☐ Always.

1.3. Even though I know I must focus on the positive side of things, I find it very hard to do.
☐ Never. ☐ Rarely. ☐ Sometimes. ☐ Often. ☐ Very often. ☐ Always.

Demographical data.

2. **Your age**: ______________________________
3. **Geographical area where you work**: ______________________________
4. **What is your specialty**: ______________________________
5. **I had contact with COVID-19 positive patients** ☐ No. ☐ Yes.
6. **My type of working contract is:** ☐ Permanent. ☐ Temporary. ☐ I was hired for this emergency.
7. **I am currently working at a** ☐Primary care. ☐ First-level hospital. ☐ Second-level hospital. ☐Third-level hospital.

8. **My job experience is** ☐ 0-1 year. ☐ 1-3 years. ☐ 3-5 years. ☐ 5-10 years ☐ 10-20 years. ☐ more than 20 years.
9. **Do you have any dependants?** ☐ No. ☐ Yes.
10. **1Are you living with a partner at the moment?**
    ☐ No.
    ☐ Yes, and he/she belongs to the healthcare environment.
    ☐ Yes, and he/she does not belong to the healthcare environment.
11. **I currently work in** ☐ Intensive care. ☐ Surgical area. ☐ Hospitalization ward. ☐ Outpatient visits. ☐ Emergency room. ☐ Other.
12. **I have personally been affected by coronavirus to the following extent:**
    ☐ I have been asymptomatic.
    ☐ I have had symptoms.
    ☐ I have been in quarantine.
    ☐ I tested positive for COVID-19.
    ☐ I was admitted in the hospitalization ward.
    ☐ I was admitted in the ICU.

Other items related to physical and psychological well-being during COVID-19 pandemic.

13. **I feel physically overloaded at work.**
    ☐ Never. ☐ Rarely. ☐ Sometimes. ☐ Often. ☐ Very often. ☐ Always.
14. **I feel emotionally overloaded at work.**
    ☐ Never. ☐ Rarely. ☐ Sometimes. ☐ Often. ☐ Very often. ☐ Always.
15. **I feel my health is threatened because of actual risks at work.**
    ☐ Never. ☐ Rarely. ☐ Sometimes. ☐ Often. ☐ Very often. ☐ Always.
16. **I feel disappointed with the work dynamics established at my job.**
    ☐ Never. ☐ Rarely. ☐ Sometimes. ☐ Often. ☐ Very often. ☐ Always.
17. **I feel unsatisfied with the hierarchical structure established at work.**
    ☐ Never. ☐ Rarely. ☐ Sometimes. ☐ Often. ☐ Very often. ☐ Always.
18. **The fact of finding myself within an unfamiliar environment and team affects my personal stability and performance at work.**
    ☐ Never. ☐ Rarely. ☐ Sometimes. ☐ Often. ☐ Very often. ☐ Always.
19. **I feel my job is not making any difference to social advancements towards resolution.**
    ☐ Never. ☐ Rarely. ☐ Sometimes. ☐ Often. ☐ Very often. ☐ Always.
20. **I am afraid of whatever may happen after this health emergency. State your level of agreement.**
    ☐ Never. ☐ Rarely. ☐ Sometimes. ☐ Often. ☐ Very often. ☐ Always.
21. **I feel I can not balance my family life because of my job. State your level of agreement.**
    ☐ Never. ☐ Rarely. ☐ Sometimes. ☐ Often. ☐ Very often. ☐ Always.
22. **I am receiving some kind of psychological support at the moment:**
    ☐ No.
    ☐ No, but I would like to.
    ☐ Yes, I was in therapy before the emergency.
    ☐ Yes, I am in therapy since the emergency began.
    ☐ I am getting other forms of psychological support rather than conventional psychotherapy.
23. **Date:** ____
